# The role of information science within the clinical translational science ecosystem

**DOI:** 10.1017/cts.2024.664

**Published:** 2024-11-27

**Authors:** Bart Ragon, Anne Seymour, Elizabeth C. Whipple, Alisa Surkis, Amanda Haberstroh, Jennifer Muilenburg, Melissa L. Rethlefsen, Erinn E. Aspinall, Jill Deaver, Nadine Dexter, Renae Barger, Nicole Contaxis, Emily J. Glenn, Elizabeth Hinton, Barbara Kern, Micquel Little, Keith Pickett, Erika Sevetson, Donghua Tao, Megan von Isenburg, Debra A. Werner, Terrie R. Wheeler, Kristi Holmes

**Affiliations:** 1 University of Virginia, Claude Moore Health Sciences Library, Charlottesville, VA, USA; 2 Integrated Translational Health Research Institute of Virginia (iTHRIV), Charlottesville, VA, USA; 3 Johns Hopkins University, Welch Medical Library, Baltimore, MD, USA; 4 Indiana University School of Medicine, Ruth Lilly Medical Library, Indianapolis, IN, USA; 5 Indiana Clinical & Translational Sciences Institute, Indianapolis, IN, USA; 6 NYU Grossman School of Medicine, Health Sciences Library, New York, NY, USA; 7 East Carolina University, Laupus Health Sciences Library, Greenville, NC, USA; 8 University of Washington, Health Sciences Library, Seattle, WA, USA; 9 University of New Mexico, Health Sciences Library & Informatics Center, Albuquerque, NM, USA; 10 University of Minnesota, Health Sciences Libraries, Minneapolis, MN, USA; 11 University of Alabama at Birmingham, Lister Hill Library, Birmingham, AL, USA; 12 University of Central Florida Health Sciences Library College of Medicine, Harriet F. Ginsburg Health Sciences Library, Orlando, FL, USA; 13 University of Pittsburgh, Health Sciences Library, Pittsburgh, PA, USA; 14 University of Nebraska Medical Center, Leon S. McGoogan Health Sciences Library, Omaha, NE, USA; 15 University of Mississippi Medical Center, Rowland Medical Library, Jackson, MS, USA; 16 Queen’s University, Queen’s University Library, Kingston, ON, Canada; 17 University of California, San Francisco, UCSF Library, San Francisco, CA, USA; 18 Tulane University, Rudolph Matas Library of the Health Sciences, New Orleans, LA, USA; 19 Brown University Library, Health and Biomedical Library Services, Providence, RI, USA; 20 University of Illinois Chicago, Library of Health Sciences, Chicago, IL, USA; 21 Duke University, Duke University Medical Center Library, Durham, NC, USA; 22 University of Chicago, John Crerar Library, Chicago, IL, USA; 23 Weill Cornell Medicine, Samuel J. Wood Library, New York, NY, USA; 24 Northwestern University Feinberg School of Medicine, Northwestern University Clinical and Translational Sciences Institute, Chicago, IL, USA; 25 Northwestern University Feinberg School of Medicine, Galter Health Sciences Library and Learning Center, Chicago, IL, USA; 26 Northwestern University Feinberg School of Medicine, Department of Preventive Medicine, Chicago, IL, USA

**Keywords:** Health sciences libraries, Clinical and Translational Science, partnership, Clinical and Translational Science Award Program Hubs, collaboration

## Abstract

Academic health sciences libraries (“libraries”) offer services that span the entire research lifecycle, positioning them as natural partners in advancing clinical and translational science. Many libraries enjoy active and productive collaborations with Clinical and Translational Science Award (CTSA) Program hubs and other translational initiatives like the IDeA Clinical & Translational Research Network. This article explores areas of potential partnership between libraries and Translational Science Hubs (TSH), highlighting areas where libraries can support the CTSA Program’s five functional areas outlined in the Notice of Funding Opportunity. It serves as a primer for TSH and libraries to explore potential collaborations, demonstrating how libraries can connect researchers to services and resources that support the information needs of TSH.

## Introduction

Translational science is focused on decreasing the time it takes to “translate” bench research into healthcare practice in order to deliver new interventions to the communities and patient populations that need them [1]. To advance this critical goal, the National Institutes of Health (NIH) has awarded Clinical and Translational Science Awards (CTSA) since 2006, enabling a network of hubs to advance clinical and translational science throughout the United States. Since 2012, the program has been supported through the National Center for Advancing Translational Sciences (NCATS) to apply translational science principles to more quickly convert biomedical research into better health outcomes [2].

Academic health sciences libraries (“libraries”) have long partnered with their constituents to support basic, clinical, and translational research and the related information needs of clinicians and patient populations. Most libraries offer a range of resources and services supporting the research lifecycle, making them natural partners for clinical and translational science. Many libraries have a rich history of working with CTSA Program hubs and other translational efforts such as the IDeA Clinical & Translational Research Network (CTR-N) [3].

In 2021, the CTSA Program Notice of Funding Opportunity (NOFO) was revised and restructured. The new announcement consists of five elements: Overview, Strategic Management, Training & Outreach, Clinical and Translational Science Resources and Pilots, and a Clinical and Translational Science Research Program [4,5]. The new structure represents an evolution of the CTSA Program to advance the science of translational science. A significant modification to the CTSA Program involves greater incorporation of open science and data science into program areas and enhanced requirements for the dissemination and implementation of translational research from discovery to improved health of the community. This translational science spectrum illustrates how each stage of translational research informs and builds upon the next to ensure that translational research leads to improvements in human health [6–8].

In 2013, Holmes et al. explored the ways that academic health sciences libraries support clinical and translational science based on the functional areas defined by the NCATS program at that time [9]. Much like the CTSA Program, libraries have experienced a significant period of evolution over the last decade, offering an opportunity to revisit, update, and reimagine their support of translational science. This article aims to revisit potential library partnerships with Translational Science Hubs (TSH) considering the evolving structure and mission of these hubs, as well as the ongoing expansion in library services over the past 11 years. For the purposes of this article, TSH includes CTSA Program Hubs and other academic clinical and translational science institutes, a wide range of NIH funding opportunities, and other programs supporting clinical translational science. Here we address areas of academic health sciences library support for each of the CTSA Program’s five functional areas contained in the NOFO. Since TSH programs and libraries are each unique in their structure and priorities, this article is intended to serve as a jumping-off point for TSH and libraries to explore potential partnerships. Partnership with libraries can help connect researchers to relevant library resources and services, support scholarly activities by TSH investigators, and promote the development of new library services and infrastructure supporting clinical and translational science. The authors also believe that the principles outlined are applicable in a variety of settings beyond TSH and can be a useful scaffold upon which to design, develop, and disseminate library programs and resources in support of biomedical research and its application to patient care and population health.

## Methods

A team science approach was utilized for this project, integrating findings from a literature review, practical experiences of academic health sciences librarians, and collaborative writing. Case studies explored several examples of successful partnerships with translational science programs. The data collected was mapped to the Clinical and Translational Science Award Program’s five functional elements outlined in the NOFO PAR-24-272 [5]. Librarians from multiple institutions engaged in project activities, discussions, and collaborative writing to share insights and identify opportunities and key factors that drive successful partnerships.

Support for translational science by libraries and librarians is well-represented in the biomedical literature. To conduct this literature review, the authors searched PubMed to identify library or informationist projects for clinical and translational research efforts and included articles that explicitly referenced library collaborations that had librarian coauthors and explicitly referenced library collaborations with their institutional TSH. Additionally, the authors searched Medical Library Association (MLA) Annual Meeting abstracts for papers and posters from MLA annual meetings 2013–2023, including those that mentioned “translational science” in the abstracts.

The literature search identified numerous opportunities for collaboration between libraries TSH that align with organizational priorities. Library partnerships often feature the development of tools and infrastructure to foster discovery and collaboration, critical components of any TSH. Assessment of information needs [11] and creation and maintenance of researcher profile systems [12] represent examples of this role. Additionally, active engagement with pilot projects to develop and create clinical and translational science personas to understand specific roles has grown over several years [13–16]. The development and use of personas contribute to a more nuanced understanding of the different roles and needs of a translational science team, further highlighting how health sciences libraries can provide support.

Libraries also frequently contribute to evaluation and assessment of research impact. Examples include use of social network analysis tools to track and visualize inter-institutional interactions of TSH programs and investigators [17], creating a systematic review core facility to support evidence synthesis research [18], and hosting library-based drop-in services for TSH core services, therefore providing a one-stop shop for support. In 2016, librarians and program staff from numerous CTSA Program Hubs identified inconsistencies in the categorization of TSH-linked publications and applied machine learning to create a structure by which publications can be classified according to their phase in the translational research spectrum [19]. In 2017, librarians partnered with a TSH to define the concept of “translational research” [20]. Librarians have also played critical roles in defining and developing models for assessing translational science impact [21–25] and many health sciences libraries regularly contribute bibliometric, social network, and funding analyses [9, 26–32].

Development of the clinical and translational science librarian workforce continues to be a critical component in the ability of libraries to meet this need on their campuses. To meet this pressing workforce development need, Cleveland et al. developed a virtual three-credit hour graduate-level course, “Genomics and Translational Medicine for Information Professionals” [33] through the Health Informatics Program at the University of North Texas College of Information. Peer learning and exchange also play an important role in the ongoing development of a translational science-minded library workforce. This often occurs through development activities and training at professional meetings. Librarians actively share and discuss their translational science partnerships and activities through presentations, posters, and conference papers given at a range of events, predominantly the MLA annual meeting, but also at clinical and translational science Program meetings, the Association of Clinical and Translational Sciences annual meeting, the Association of American Medical Colleges Group on Information Resources meeting, and American Medical Informatics Association meetings. These presentations cover a wide range of topics, including library support for clinical research scholars [34,35].

## Library partnership activities

**Figure 1.**
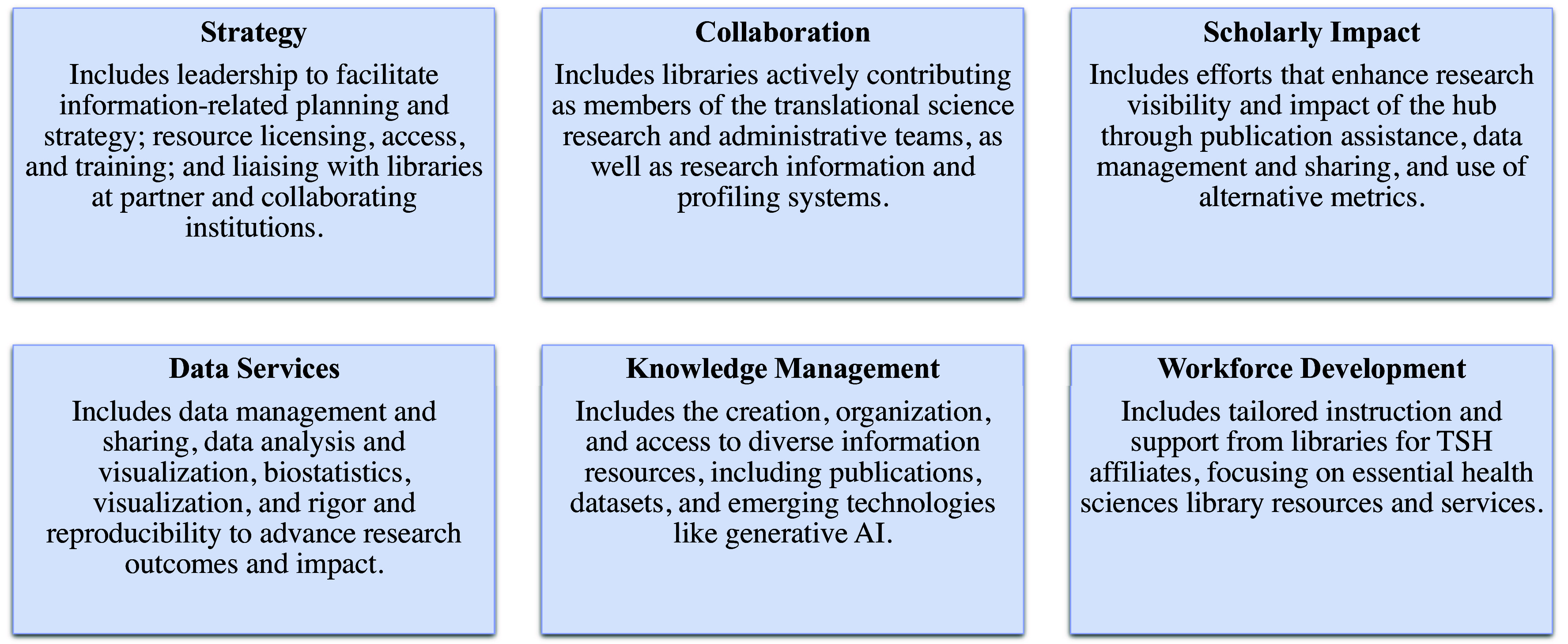
Key topical categories for library-Translational Science Hubs partnership.

Library partnerships in the literature as well as the services and goals of health sciences libraries align with critical functional areas described in the CTSA Program NOFO. These are described in more detail in the following section and frequently intersect such topics as Strategy, Collaboration, Scholarly Impact, Data Services, Knowledge Management, and Workforce Development (Fig. 1). It is important to note that this is not an exhaustive list. Indeed, each TSH and its associated library (or libraries) bring unique perspectives, resources, and expertise, and it is, therefore, worthwhile to leverage these ideas as inspiration for TSH and their libraries to generate meaningful local ideas for collaboration and partnership.

### Overview (Element A)

The Overview element requires that TSH applicants define the overall vision and strategic goals for the hub and with the national CTSA Program along with related strengths for the research environment, including descriptions of the team, their high-impact achievements, and the expected impact on science and public health [4]. Many libraries provide scholarly impact services that use bibliometrics and alternative metrics to illustrate the strengths and impact of clinical and translational science by measuring the dissemination and use of scholarly outputs. Library expertise can support TSH by assessing and visualizing the connections of scholarship between partner and collaborator institutions, as well as the hub’s impact on scholarly outputs [31,32]. These scholarly impact metrics can be used to demonstrate the value of clinical and translational science over time and can assist in the creation of evaluation metrics and references that support grant application narratives and funder reporting requirements. Librarian-led literature reviews on key topics can also strengthen application narratives.

### Strategic management (Element B)

The Strategic Management element requires a description of operations and infrastructure needed to support the TSH [4]. Strategic management defines governance, leadership structure, integration within the CTSA Program national network, and coordination of resources and programs. Libraries can support strategic management by participating as a member of the TSH’s leadership/governance to oversee information-related planning and strategy; assisting with software and resource licensing, access, and training; providing liaison services for research support; and acting as a liaison between libraries at partner and collaborating institutions. Librarians have contributed to strategic planning and practical solutions to support adaptive capacity at hubs, and critical activity in the context of COVID to support disaster planning [36–41]. Central to the role of libraries are the requirements for ongoing assessment and program evaluation, and dissemination and implementation (D&I) activities. Libraries can actively support D&I goals of the hub through collaboration on resources and services to support D&I and directly support dissemination by providing guidance on scholarly communication practices, compliance with federal funders’ open access and data sharing mandates, and open science strategies. In addition, many libraries provide services and resources, see Table 1 as an example, to support assessment and program evaluation, including faculty profile systems, collaboration analyses, data visualization and dashboard tools and services, and scholarly impact metrics. The TSH and library sit within the Learning Health System environment, offering additional opportunities for coordination, support, and resources to support the entire enterprise.

**Table 1.** Strategic management use case example

*In a multi-hub collaboration led by Northwestern University’s Galter Health Sciences Library, librarians and researchers worked together to develop clinical and translational science Personas, a portfolio of nineteen evidence-based profiles based on roles across the ecosystem of translational research, spanning basic research to public health, including two patient profiles* [*14,15*]. *These representative profiles are designed to assist TSH in evaluating the resource and service needs for supporting the translational science workforce and community, including development of software, tailored educational and communication materials, understanding key perspectives and needs, providing context to better support workforce development, and targeted guidance for such pressing issues as institutional considerations of the National Institutes of Health’s (NIH) Data Management and Sharing Policy [35].*

### Training & outreach (Element C) – Workforce development for clinical research staff professionals module and community and stakeholder engagement research module

The Training & Outreach element includes two distinct modules: (1) workforce development for clinical research staff professionals and (2) community and stakeholder engagement [4]. Training and outreach are foundational components of most health sciences library services, providing a unique opportunity for TSH to leverage an existing, well-developed strength of health sciences librarians.

### Workforce development for clinical research staff professionals

The workforce development for clinical research staff professional’s module encompasses a variety of educational activities, including workshops, seminars, online classes, and other opportunities that support professional development, with an emphasis on early career researchers and team science. Training and outreach are core functions of libraries and are easily translatable to the clinical and translational science environment. Libraries can partner in the creation of clinical and translational science information guides, information discovery through databases like PubMed, training, and support for systematic reviews and evidence syntheses, data management planning (see Table 2 example), and the use of scholarly repositories to support preservation and dissemination of research. In addition, many libraries offer resources and expertise to support instruction on best practices in data sharing, data science skills such as coding, the science of team science [42], open science best practices, rigor and reproducibility in science, and knowledge and expertise supporting IRBs (Institutional Review Boards) and/or the IACUC (Institutional Animal Care and Use Committee). Libraries support mentorship through workshop offerings and resources. Librarians have also developed and published code that uses structured data to automatically generate training grant tables illustrating mentor-mentee relationships in NIH format [43]. Academic health sciences libraries are increasingly offering artificial intelligence (AI) training and resources, including research information discovery tools, prompt engineering, ethics, copyright, literature reviews, and citation of use [44–48].

**Table 2.** Training and outreach use case example

*In 2023, the University of Virginia Health Sciences Library partnered with the integrated Translational Health Research Institute of Virginia (iTHRIV) to develop training and resources for researchers. This initiative aimed to support research teams in preparing for and implementing the new National Institutes of Health (NIH) Policy for Data Management and Sharing (DMSP). A dedicated website, hosted by the library and accessible through the iTHRIV Research Concierge Portal, offers policy guidance, including links to NIH-hosted information, resources from other institutions, and newly developed UVA templates and suggested proposal language [36,37]. This collaboration between iTHRIV and the Health Sciences Library provided institutional leadership, facilitating communication and engagement with researchers by leveraging the long standing relationship between the library and iTHRIV.*

### Community and stakeholder engagement research

The community and stakeholder engagement research module includes support for building effective teams, dissemination of research, and diversification of representation in research teams and participants to create meaningful connections between research findings with individual communities, especially rural, minority, and underserved populations. Academic health sciences librarians are critical partners in understanding and addressing bias in the research lifecycle and can acquire and create information resources on anti-racist practices in primary and secondary research, data, publishing, and information searching [49,50]. Additionally, librarians have considerable expertise in training a wide range of roles on how to find and apply credible health information and how to adopt the FAIR principles (Findable, Accessible, Interoperable, and Reusable), ensuring researchers, members of the public, and community-based health organizations have access to the best information from reliable sources. Academic health sciences libraries can also be partners in community engagement through their relationships with public libraries and other community organizations by providing health information to consumers, as well as educating public librarians in consumer health information best practices. Academic health sciences librarians are skilled in the creation of online information guides, best practices in licensing and access to resources, and support of citizen science, all of which can advance goals around community and stakeholder engagement.

### Clinical and translational science resources and pilots (Element D) – Resources and services module, clinical and translational science pilot module, and data science module

The Clinical and Translational Science Resources and Pilots element includes three modules: 1) resources and services, 2) clinical and translational science pilots, and 3) data science [4].

### Resources and services

The resources and services module may support needs across a broad range of areas, including biostatistics and research design, data science and informatics, research recruitment, provision of core lab equipment, and general training opportunities. Libraries provide support throughout the entire research lifecycle. Many libraries contribute to TSH biostatistics needs with resources and services, including training workshops, statistical software licenses, and collection resources such as journals, books, and reference materials. This includes support for data collection tools like REDCap and other platforms, data visualization and dashboards, as well as utilizing programing languages such as R and Python (see Table 3) for data analysis and reporting [51,52]. Libraries regularly provide instruction for data management, federal funder open access mandates, rigor and reproducibility, and best practices in research integrity and responsible research [53–55]. Librarians act as data governance custodians and can provide regulatory support for protected data sets inside a secure data enclave and collaborate with IRBs to ensure data protection and authorized access [56].

**Table 3.** Clinical and Translational Science resources use case example

*The University of Pittsburgh Health Sciences Library System has actively partnered with their campus to offer training, resources, and services to* clinical and translational science *researchers for many years. These offerings cover a broad range of topics such as informatics and data science, scholarly publishing, compliance, research integrity, and FAIR (Findable, Accessible, Interoperable, Reusable) best practices. Recognition for librarians’ teaching contributions within the CTSI Responsible Conduct of Research (RCR) Center have been recognized with secondary appointments with the University’s Clinical and Translational Science Institute (CTSI).*

### Clinical and translational science pilots

The clinical and translational science pilot module provides support for new and innovative projects. Libraries provide a variety of research support services that can be leveraged for pilots. Libraries support literature searches to ensure that translational science projects are aware of relevant scientific evidence in the scholarly record. Libraries may also provide guidance on the dissemination of research products, including journal selection, data description, repository selection (including use of local institutional repositories), and the use of information management and synthesis tools. Because facilitating the research process is a core aspect of libraries in the learning health system, many library initiatives may be of interest to pilot project investigators, including the development of tools for data discovery and identification of potential collaborators.

### Data science

The data science module provides an opportunity to leverage the resources and expertise of the hub in data science, informatics, and open science to advance clinical and translational science and enhance the quality of research. This includes a variety of research activities, resources, and services, including data management and sharing, cohort discovery via i2b2 and Leaf, extraction of Electronic Health Record data, computable phenotyping, data description and discovery, privacy, and security. Given the strong connection from data science and informatics to information science, this is a particularly strong area of collaboration between libraries and TSH. Librarians regularly provide training and consultation services across a wide range of data science and informatics topics and often serve in leadership roles [57] or as co-investigators on projects and major initiatives. They also can support researchers through the creation of information guides and delivery of other resources. Many libraries have developed expertise and provide extensive training and support in metadata, federal funder open access and data sharing mandates, data ethics, best practices for reproducibility, and open science practices and tools. Libraries have invested in training or tools to support bioinformatics and data science services [20,58–60]. Libraries are also playing important roles to meet the urgent need for training, support, and responsible use of AI tools.

Libraries play a central role in research data sharing and discovery [61] as well as initiatives to support meaningful data metrics[62] and incentivize good data practices. Libraries have developed specialized data catalogs to facilitate discovery of existing institutionally created data sets that can be located/mined for secondary use [63,64]. These catalogs are designed to encourage collaboration and cross-disciplinary discovery. Libraries also develop and deliver campus resources [65] and repository infrastructures to support funders’ data-sharing mandates and support FAIR principles best practices and equitable access to knowledge. Examples of this include institutional repositories [66,67] and partnerships on repository projects with domain-specific repositories or generalist repositories, such as the NIH Generalist Repository Ecosystem Initiative [68–70]. Libraries are also frequent and active collaborators in the development, implementation, and/or support of institutional profile systems and data (see Table 4) to facilitate collaboration [71,72]. This work builds upon library expertise and resources and supports a range of efforts beyond researcher profiles that support team science.

**Table 4.** Clinical and Translational Science resources use case example

*Weill Cornell Medicine librarians and administrators collaborated with the Informatics team of Weill Cornell Medicine’s Clinical & Translational Science Center to develop ReCiter [50,59]*, *an open source publication management and reporting system. ReCiter identifies individual-authored publications in PubMed by harnessing institution-specific identity data. Its companion system, ReCiter Publication Manager [52] allows everyone at Weill Cornell to generate reports and publication lists for all faculty, students, alumni, and residents. It also empowers these end users to generate bibliometric reports for individual full-time faculty which summarize data from iCite [51,52]*, *NIH’s bibliometric reporting tool.*

### Clinical and translational science research program (Element E)

The Clinical and Translational Science Research Program is intended to be a discrete research project, with the intent that project innovations are generalizable in other translational science settings. The library research support services described in the preceding sections – such as literature searching, data management support, evidence synthesis, data visualization, and coding instruction – can serve to support this project through the research lifecycle. Furthermore, because the research project(s) described in Element E may have more specific needs, TSH is encouraged to contact your library about other resources or services that may be available.

Ultimately, there are several examples of points of successful partnerships between TSH and libraries – with many more potential areas of collaboration and capacity left to explore. Several examples inspired by the NOFO as well as currently underway at TSH are highlighted in Table 5.

**Table 5.** Library contributions and partnerships that can support local Translational Science Hubs (TSH) work

	WORKFORCE DEVELOPMENT	STRATEGY	COLLABORATION	SCHOLARLY IMPACT	DATA SERVICES	KNOWLEDGE MANAGEMENT	RELEVANT ELEMENT(S)
Serve as active members of the leadership team and advise on information planning and strategy; provide partnership and support for the TSH collaboration, coordination, and communication infrastructure		•	•			•	**B**
Act as a liaison between libraries at partner and collaborating institutions		•	•	•			**B**
Contribute to CTSA consortium activities, including scientific, training, governance, workgroups, etc.	•	•	•	•			**AC**
Manage faculty profile system and/or the information resources used to populate these systems	•	•	•	•	•	•	**B**
Evaluation services and resources Assessment and visualization services, including collaborative activities, interpersonal or inter-organization partnerships, areas of strength Demonstrate research impact through bibliometrics, scientometrics, alternative metrics, and data citations Provide guidance and text for metrics and impact statements to include in the grant narrative, such as citations resulting from previous translational initiatives, examples of knowledge translation, open science highlights, and descriptive text about collaboration impact Serve as a member of the evaluation and continuous improvement team	•	•	•	•	•	•	**ABCDE**
Provide resources and support for marketing and dissemination strategies	•	•	•	•			**BC**
Resources, services, and training for investigators, trainees, and staff (e.g., consultation services, resource guides, workshops, programing and invited speakers, etc.) Data science and informatics methods and tools Biostatistics methods and tools, statistical software (e.g., R, Python, SAS, SPSS), rigorous and reproducible research Artificial Intelligence AI information discovery tools supporting research (including prompt engineering, ethics, etc.) Scholarly communications (e.g., scientific writing, scholarly publishing, citations, systematic and scoping reviews, etc.) Open Science practices and tools including the FAIR principles Information discovery, management & synthesis tools (PubMed, Covidence, Rayyan, Zotero, etc.) Team science, collaboration, contributor roles, etc. Data management and sharing policies, practices, and solutions (e.g., use of DMPTool; elements of a NIH Data Management and Sharing Plan, data curation, metadata best practices, data reuse, etc.) Research integrity (e.g., responsible conduct of research, data ethics, responsible use of AI tools, understanding and addressing bias in the research lifecycle, etc.) Data discovery and use of data for secondary analysis	•	•	•	•	•	•	**BCDE**
Provide guidance and expert services for clinical research staff (e.g., instruction, consultation support, support and assist with the development of curricula) on relevant topics such as research rigor and reproducibility, IRB and/or IACUC, team science, data management planning, open science, metadata, critical appraisal, etc.	•		•			•	**C**
Repository and data catalog resources and services Compliance with funder research data sharing requirements Support use of institutional, domain, and generalist repositories Assist with the dissemination of information including journal selection and online repositories Implement and host Data Catalogs to facilitate the discovery and use of existing data for secondary analysis Share and host datasets, presentations, posters, and other research objects. House biostatistics seminar recordings and training materials in a collection in the institutional repository Provide support and guidance on data governance and management of restricted data enclaves (regulatory support)		•	•	•	•	•	**BCDE**
Support vendor communications and licensing of software or other information resources; consult about the licensing of resources for institutional partners, including navigation of legal aspects of resource sharing		•	•		•	•	**BD**
Training and workforce development Consultation and training for online learning platforms Provide space for collaboration and training events for the translational science workforce Support training and educational activities (e.g., educational seminars, workshops, e-learning courses, experiential opportunities) Partner on the development of innovative or improved approaches to the dissemination of educational content (e.g., use of interactive online platforms to optimize the end-user’s experience) Operationalize online learning platforms, including a plan to sustain the platform independent of direct CTSA funding support	•		•			•	**BCD**
License, manage, and provision scientific software via an institutional software hub			•		•	•	**BD**
Meet with pilot teams early in their work to provide guidance and resources that support good data practices, establish tracking of research outputs, and support dissemination strategies			•	•		•	**C**
Contribute to onboarding activities; assist with integrating new employees into the hub	•	•	•				**BC**
Develop user agreements or licenses to maximize the adoption, implementation, or use of educational content, innovations, tools, data, and software developed by the local TSH by other organizations	•	•	•	•	•	•	**CD**
Support communitybased research and equitable access to resources Explore and help license resources that advance learning and practice for culturally competent, community-based research Facilitate collaboration with Community Based Health Organizations and other members of the community with available resources and services Partner on community engagement opportunities to inspire participation in research, via community health information activities, basic research skills, citizen science; consider collaboration with public library partners in the community Provide support to investigators for making data and research products accessible and discoverable to the community Develop and deliver physical and virtual exhibits to highlight community history on health topics Provide or partner on training and resources to support open equitable access to research from the TSH Identify culturally and linguistically appropriate strategies in the literature to support underrepresented populations for CTSA education and training activities	•	•	•	•	•	•	**BCD**

**Library contributions and partnerships to support local Clinical and Translational Science efforts.** Library partnerships and activities to address key topics and needs at Translational Science Hubs (TSH). Topical categories of Strategy, Collaboration, Scholarly Impact, Data Services, Knowledge Management, and Workforce Development (Fig. 1) are noted in columns, with intersections indicated with dots in the corresponding category. Relevant elements from the CTSA NOFO are shown in the right-most column (A: Overview, B: Strategic Management, C: Training and Outreach, D: Clinical and Translational Science Resources and Pilots, E: Clinical and Translational Science Research Program).

## Discussion

The integration of academic health sciences libraries’ specialized expertise and support activities into the TSH is a strategic alliance aimed at bridging gaps between research innovation and practical application in the learning health system. The breadth and depth of the examples discussed in this work and listed in Table 1 underscore the significant contributions of libraries in supporting clinical and translational science, highlighting their role in enhancing research visibility, developing a skilled and trained research workforce, promoting interdisciplinary collaboration, and increasing compliance with evolving research mandates.

Libraries have proven instrumental in leveraging bibliometrics and alternative metrics to showcase the impact of clinical and translational science, including visualizing the connections of scholarship across institutions and illustrating the dissemination and utility of translational science scholarly outputs. Furthermore, librarian expertise in scholarly communication, knowledge of publication open access and data management and sharing federal mandates, and implementation experience with open science initiatives (leveraging the FAIR principles), can contribute to a foundation of robust resources and expertise, governance perspective, and operational experience to successfully support and sustain the work of the TSH. This involvement ensures that information-related strategies are aligned with the overarching goals of clinical and translational science, facilitating seamless integration within the CTSA Program or other translational science national network. Expertise may vary widely between academic health sciences libraries. In certain cases, particularly those involving data management and governance of human subject research, libraries may not be the most suitable source of credible information. In such instances, TSH and libraries can explore additional partnerships, such as compliance or information technology units, to support translational science needs. As previously mentioned, the research lifecycle framework can be a valuable tool for identifying additional opportunities for collaboration and enhancing support for clinical and translational science [10].

The provision of specialized resources and services further exemplifies the critical role of libraries in clinical and translational science. As information technologies advance, the scope of traditional knowledge formats, such as books and journals, has expanded to include datasets, code, and other emerging forms of information. Many libraries and librarians have adopted new roles in supporting these areas and by offering support for research tools such as REDCap, R, Python, SAS, and SPSS, and providing instruction in data management and compliance with open access mandates, libraries facilitate rigorous and responsible research practices [73–81]. Furthermore, library expertise in evidence synthesis enables them to lead research teams in using best practices and developing research protocols that directly ensure the replicability of these evidence reviews. Moreover, their involvement in good data practices and long history of providing research consultation services and training underscore a commitment to ensuring high standards of research integrity and reproducibility. Furthermore, libraries can act as a liaison between libraries at partner and collaborator institutions, ensuring researchers and administrators have access to their institutional resources supporting translational science, and reinforcing active connections with partners.

The opportunity to consider clinical translation science in the context of the TSH offers new opportunities for libraries to collaborate with TSH and co-develop training and resources to meet emerging needs in an efficient and effective manner. It is important to recognize that these partnerships can start and become productive even before formal funding mechanisms are in place. Early engagement during the proposal development phase is a great way to raise awareness of existing library services and resources that support the translational science spectrum. This early engagement can also pave the way for deeper and long-lasting partnerships between libraries and TSH.

Ultimately, the multifaceted support provided by libraries directly reflects the priorities of the TSH and is critical to the success of clinical and translational science. Libraries’ contributions across the functional areas demonstrate the *potential translational synergies* of this collaboration, as well. As TSH continues to evolve, the sustained inclusion of health sciences librarians will be crucial in ensuring that scientific discoveries are efficiently translated into tangible health benefits for communities.
